# Design and Implementation of Intelligent Learning System Based on Big Data and Artificial Intelligence

**DOI:** 10.3389/fpsyg.2021.726978

**Published:** 2021-11-11

**Authors:** Yawen Yang

**Affiliations:** Academy of Arts, Southwest Minzu University, Chengdu, China

**Keywords:** multimedia teaching, multimedia technology, information management system, module design, big data, information technology

## Abstract

In order to improve the level of education development in remote areas of China, help minority schools to better play the advantages of multimedia network teaching resources provided by the state, and further improve the level of multimedia network management, a multimedia network teaching management system is studied and developed. The teaching information and business process of the school is analyzed, the division of the structure of the film and television multimedia information management system is completed, and the overall structure, functional structure, and database of the system are designed; Tornado technology, NoSQL database technology, jQuery technology, and Ajax technology are used to complete the development of the system. The results show that the real system can provide a highly automated platform for the school multimedia network-teaching management. The system makes the teaching staff work more efficiently and accurately. The results show that the system studied has a significant effect on the multimedia teaching of minority films and television. Using the characteristics of the combination of pictures, text, sound, image, and shadow, the knowledge can be shown to students intuitively, which is helpful to relaxed and happy learning of students and stimulate their desire to learn. Multimedia teaching has become an important teaching assistant tool. This exploration can provide theoretical support for the design of the network teaching information management system based on artificial intelligence, decentralize the complex network environment, and carry out decentralized management.

## Introduction

Ethnic minority areas are vast in territory, inconvenient in transportation, blocked in information, and poor in quality of teachers. Limited educational resources are lack of integration, scattered in strength, overlapping, and wasting seriously. Various forms of education and school-running types lack communication, high-quality resources are difficult to share, and the quality and efficiency of teacher training are difficult to guarantee (Ying, 2017). However, the big data technology can open up new space for minority teaching and maximize the sharing of teaching resources, and it has the characteristics of huge capacity, wide variety, extremely fast acquisition speed, strong variability, and relatively low degree of authenticity, so it is widely employed in teaching. Fully utilizing modern multimedia network education means and adopting flexible and diverse modes are conducive to breaking the space-time barrier. Utilizing and sharing high-quality resources in developed areas will play a very positive role in realizing the optimal allocation of resources and improving the overall level of development of ethnic education, especially in accelerating the development of basic education in ethnic areas (Visser et al., 2017). The application and the development of artificial intelligence (AI) technology have been widely used in various fields of society and promoted the comprehensive reform of quality education. Compared with the traditional education mode, the network education of AI technology has rich teaching resources and information. In teaching, the traditional teaching mode is not enough for students to deeply learn detailed knowledge. Therefore, the network teaching resource information-integrated management system needs to be designed to promote the network teaching mode.

A Nationality Teachers College is far from the central city, located in a town in Ganzi Tibetan District, where traffic and information are very blocked. It undertakes the historic task of “stabilizing Tibetan well-being” and training all kinds of talents for Tibetan area (Hwang and Park, 2016). In order to give full play to the advantages of multimedia network education in training talents and cultivate more innovative talents in Tibet, it is necessary to seize the commanding heights of network education. Since 2000, the school has invested tens of millions of yuan to build a 100-M campus computer network connecting each classroom, office, laboratory, student dormitory, and the family of a teacher, and set up various media network classrooms, interactive multimedia network language laboratories, and multimedia network teaching computer rooms (Mershad and Wakim, 2018). With the establishment of these hardware facilities, a completely new and modern multimedia network teaching environment will be constructed. The application of multimedia technology enriches the original single teaching mode of the school and greatly improves the quality of the education and teaching and teaching management level, but there are also many problems. The technology of film and television multimedia is studied, and the information management system of film and television multimedia network teaching is designed by referring to the development of similar systems. The construction of these hardware facilities will build a completely new modern multimedia network teaching environment, enrich the original single teaching mode, and greatly improve the quality of the education and teaching and teaching management level.

In recent years, colleges and universities have paid more and more attention to the utilization of information technology to manage multimedia teaching resources, which aims at making better use of the Internet technology, improving the level of information management, and emphasizing on solving the problems and difficulties caused by the diversity of ethnic minority students and the diversity of their learning levels. At present, in the field of the multimedia network teaching for minority films and television, certain deficiencies, such as the presence of appropriate information management system, have led to the difficulty in information management of multimedia network teaching. In addition, most of the multimedia network teaching has not been rationally regulated by using information technology. At present, there are still some shortcomings in the minority film and television multimedia network teaching system, such as the imperfection of information management system and the lack of close links between various links, which will lead to the multimedia network teaching restricted in information management. In addition, most multimedia network teaching still stays at the application level of classroom teaching, and there is no reasonable regulation and management through information technology. Therefore, further research is needed to break through these limitations. Multimedia network teaching information management system of minority film is an important part of practical teaching. It is not only an effective extension of professional skills teaching but also a necessary means of innovative quality teaching development. Multimedia teaching, as an information platform to promote the harmonious development of bodies and minds of students, plays an important role in improving employment ability, social experience, and team consciousness of students. To sum up, the problems existing in the current network teaching information management system are summarized through the investigation. Aiming at the above problems and corresponding needs, a network teaching information management system based on meta heuristic algorithm is innovatively designed and developed. The algorithm comparison proves the effectiveness of the designed system. The research results can provide practical value for the construction of teaching information management system.

## Literature Review

With the rapid development and wide application of computer technology, computer network technology, multimedia, and communication technology, all kinds of schools at all levels have set up campus network and multimedia network classrooms, constructed multimedia network teaching environment, and carried out research and experiment of multimedia network teaching. Guo et al. (2020) proposed a digital rights management system based on blockchain. The system includes a new network architecture for sharing and managing online education multimedia resources based on public and private blockchain, and three specific smart contract schemes for recording multimedia digital rights, secure storage of digital certificates and non-intermediary verification. The software should be compiled into a system and run in a unified manner on various campus network platforms. Abdulrahaman et al. (2020) pointed out that, for the application of multimedia in teaching, in addition to text and image, the existing tools also include multimedia components, such as audio, video, and animation. The conclusion is that most multimedia solutions for teaching and learning are aimed at the user audience of teaching content and solutions on topics of interest, and the successful use of different multimedia tools on different target groups and topics can be attributed to the technologies and components embedded in their development. The software is managed and maintained by relevant functional departments, such as schools, teaching departments, finance departments, and network management centers. The same kind of data reported are inconsistent, the work efficiency is low, and the management level has not formed a joint force. How to integrate the software together to bring benefits into play or develop a set of multimedia network teaching system software has become a realistic choice for schools (Tezer and Çimşir, 2017). Wang (2021) believed that mixed media and organizational innovation have become very obvious visual technologies, and have been used to changing performance strategies and improving education quality. Real teaching practice is of great significance for schools and universities to cultivate extensive brain science progress in health, ability, and substitutes. Because of this, it is naturally very crucial to use interactive media and network innovation to improve the actual training scene. Therefore, it is necessary to make a careful demand analysis and develop a multimedia network teaching management software system accordingly. It not only creates teaching scenarios and strengthens the intuition and vividness of teaching but also breaks through important and difficult points. It organically integrates classroom teaching and greatly improves the efficiency of classroom teaching.

Zhang et al. (2021) believed that if the multimedia teaching management system is successfully developed, it can break through the limitations of students in the classroom, and learning of students will not be affected by time and space. Regarding the teaching mode and teaching design, Huang (2021) emphasized that the overall improvement of teaching and learning experience of teachers and students urges teachers and students to break away from traditional teaching concepts and modes and achieve two times the result with half the effort. The technical advantages of AI technology in using the virtual reality teaching method in digital media art creation were given. Based on the network, educational resources and information are transmitted. It mainly combines multimedia technology, adds multimedia functions to the system, disseminates various kinds of media information through the system, processes media information, and shares information to form an idealized set of multimedia network teaching environment. At the same time, Han and Yin (2021) started with the development of the multimedia teaching platform, analyzed performance of students before and after using the system, investigated satisfaction of students with the system, and obtained the impact of the system on English learning motivation of students. It can also provide a set of effective teaching management tools for teachers. Ding et al. (2020) proposed that teachers can choose teaching methods, including synchronous teaching and asynchronous teaching. When students and teachers are online simultaneously, they can carry out synchronous teaching. When only a student or a teacher is online, asynchronous teaching can be chosen. For the questions raised by students, after teachers logging on to the system, the corresponding prompt box will pop up in the system interface, prompting students to send messages. Li (2021) analyzed the feasibility of applying multimedia technology to school music education and the auxiliary function of music education, and proposed the intelligent learning characteristics of a deep learning algorithm to monitor the teaching process of music education and analyze the process quality. In the process of learning, if there are any questions, students can ask other students or teachers for answers, which can improve the efficiency of learning of students learning. According to the research of the current scholars, scholars have made in-depth research on multimedia tools, teaching methods, and other aspects. Through multimedia teaching, the structure of a teaching classroom, the teaching effect, and the quality of teaching have been effectively improved. However, there is still a lack of research on the development of the multimedia film and television network teaching system in remote and backward areas. Based on the needs of ethnic minorities, a multimedia network teaching information management system is developed.

Massive research and practice show that the success or failure of the application of management information system in China depends not only on the hard environment such as technology, capital, Internet system, application software, and software implementation but also on the soft environment such as enterprise management foundation and cultural heritage, which often play a more crucial role. Management information system is a man-machine management system. It can play a better role only in enterprises with smooth information flow and standardized management.

## Materials and Methods

There are various development tools used in the development of multimedia teaching management system of film and television.

### Tornado Technology

The Tornado framework is an open-source Web server framework. People familiar with Python language and interested in server application development are well aware that the Tornado framework was originally developed for real-time information service based on FriendFeed, a social aggregation website, to serve social applications (Wu and Tai, 2016). At first, the Tornado framework is created for social applications: Tornado is an ideal lightweight web framework. It has a few modules; the most important module is the web module, and the other modules are tool based. According to the research by Dascalu et al. (2016), the bottom core module includes three parts: http connection, iostream, and ioloop. Basalyga et al. (2021) studied the wall time difference between the real-time data enhancement method and convolutional neural network training for tornado prediction using pre-enhanced data. Fried Feed and other Tornado master developers are mainly based on Nginx or Apache agents when using them.

Tornado’s HTTP service is incomplete now. Using Tornado can easily construct various types of web servers. Now, we start with the HTTP server to see its implementation, call user-defined processing methods, and write the response data to the client socket, which are the three core modules of the Tornado server.

Tornado significantly differs from mainstream Web server frameworks (including most Python frameworks). It is a non-blocking server and is quite fast. Thanks to its non-blocking method and the use of epoll, Tornado can process thousands of connections per second, so it is an ideal framework for real-time Web services.

### NoSQL Database Technology

The relational database is based on the relational model. The relational model can express all kinds of relations between entities in the real world. The relational database stores data in different tables, which increases speed and improves flexibility. Schwabe et al. (2016) suggested that the standard framework of “LAMP” and “LNMP” in the industry development website M represents the MySQL database. Specifically, the relational database is not suitable for dealing with the following problems: first, writing large amounts of data; second, applying when fields are not fixed; third, responding quickly to simple queries; fourth, changing the structure of tables with data. The overall framework of NoSQL is divided into four layers from bottom to top, including data persistence, data distribution model, data logical model layer, and interface. The layers complement one another and coordinate work.

NoSQL generally refers to the non-relational database. It corresponds to the traditional relational database. Its appearance solves many problems that relational database cannot. The NoSQL database plays an important role in the development of the mobile Internet (Szumacher et al., 2016; Al-Ariki and Swamy, 2017; Pei et al., 2017). Although NoSQL database is only used in specific fields and it does not need to do the complicated calculation, NoSQL database well makes up for the shortcomings of the previous relational database. The strengths of NoSQL database include.

#### Easy Data Decentralization

Relational database is based on Join, so relational database must store data in the same server, which is not conducive to data decentralization. While NoSQL database does not support JOIN operation, data are designed independently, and it is easy to disperse data to multiple servers, thus reducing the amount of data on each server. It makes it relatively easy to read and write large amounts of data.

#### Flexible Data Model

According to Lech et al. (2016), the NoSQL database does not need to establish fields for each data to be stored, and does not need to formulate the attribute type, length, and other related information of each field, which can store a customized data format at any time. The data type corresponding to each field changes with the change of the storage data type, so the NoSQL database has a flexible data type.

#### High Availability

The NoSQL database has many advantages when it does not affect performance so that it can easily implement a high availability framework. The so-called high availability framework is that the database can be expanded horizontally or vertically with the increase of business, but it does not affect the performance of the system.

NoSQL does not refer to a specific data management system, which is only a concept. The NoSQL database can be divided into many types according to the storage model and characteristics of data. NoSQL data in the mainstream are timely as follows. First, column storage: including Hbase, Cassandra, and so on. The column storage database stores data by column. Column storage is characterized by convenient storage of structured and semi-structured data. Second, document storage: The document database is widely used in modern LBS (location-based service) system. A document database is usually used to store the business information in the APP. Third, key value storage: including Redis, Voldemort, and so on (Zhang et al., 2016). The key value database is usually used to store hot data in the system. That is to put the data frequently accessed by the system in memory to improve the response speed of the system. The database stored by the key value will use a hash table.

### jQuery Technology

With the continuous development of front-end technology, many developers have fully applied the idea of software reuse in software engineering to the development of technology and encapsulated many rich functions, and jQuery is one of the representatives. jQuery is another excellent JavaScript library after prototype, jQuery2.0, and the subsequent versions no longer support IE6/7/8 browsers. The core idea of jQuery is to provide Ajax interaction for websites. The jQuery module can be divided into three parts: entry module, underlying support module, and functional module. In the construction of the jQuery object module, if a selector expression is passed in when the constructor jQuery() is called to create a jQuery object, the selector Sizzle (a CSS selector engine implemented in pure JavaScript to find the set of elements matching the selector expression) is called to traverse the document, find the DOM elements matching it, and create a jQuery object containing these DOM elements. The display of jQuery is not affected by JavaScript that is being disabled. There is a situation that if Flash cannot be loaded properly, then some pages cannot be displayed properly. Of course, this situation will not occur on jQuery. Using the MIT license agreement, jQuery loads faster. Many search engines regard page loading time as a factor of SEO (Search Engine Optimization). Zhanikeev (2020) noted the similarity between DOM elements and tuples in datasets in web applications, and suggested borrowing jQuery symbols for dataset processing. jQuery files are stored separately from Web pages, which allows developers to optimize code centrally. At present, the main functions of jQuery are as follows.

#### Response Event

By introducing jQuery, developers can ignore browser compatibility issues and handle events more easily, which obviously speeds up the development efficiency of programmers, concentrates on business logic, and saves unnecessary debugging browser compatibility issues.

#### Change the Content of the Page

By using the powerful API (Application Program Interface) library provided by jQuery, pages, including content, pictures, forms, etc., can be easily modified.

#### Asynchronous Interaction With the Server

The whole set of Ajax function library provided by jQuery facilitates developers to develop applications of asynchronous interaction, frees developers from the process of writing asynchronous algorithms, and concentrates more on business processing.

#### Add Animation to Pages

jQuery provides a library of related functions instead of JavaScript, which greatly simplifies the workload of adding animation to pages (Nie et al., 2016). Different animation effects can be provided by defining different parameters.

#### Access to the Part of the Page Framework

This is one of the main tasks of the jQuery model. jQuery function facilitates developers to obtain a node in the page, rather than the information of the whole page so that the information loaded first can be realized and the experience of the user improved.

#### Simplify Common JavaScript Operations

jQuery provides many additional features to simplification operations.

The core features of jQuery can be summarized as follows. It has a unique chain syntax and a short and clear multifunctional interface; it has an efficient and flexible CSS selector, and can expand the CSS selector; it has a convenient plug-in extension mechanism and rich plug-ins. jQuery is compatible with various mainstream browsers, such as IE 6.0+, FF 1.5+, Safari 2.0+, and Opera 9.0+.

### Ajax Technology

Ajax (Asynchronous JavaScript and XML) refers to a web page development technology for creating interactive web applications. Ajax technology provides a new way of front-end and back-end data interaction. It does not need to refresh the page and does not block the page execution process. It asynchronously requests to obtain and interact data. Ajax reduces unnecessary requests and network load by exchanging a small amount of data between the server and the application server. Ajax enables asynchronous updates of web pages. Traditional web pages (which do not use Ajax) must reload the entire page if they need to update content. Initially, it just sends a request asynchronously through Ajax, and the data are a simple user name, so it just sends it directly according to the string. Later, more and more data needed to be sent by Ajax, such as a whole form, when a set of rules is needed to describe more complex data. Then, the JSON data description format is created based on JavaScript data type, which can describe complex data very simply. At the same time, it is independent of language, so it can be used in multiple languages. At present, Ajax technology is an indispensable skill for front-end developers, and, now, the mainstream front-end JS framework also integrates the excellent ideas of Ajax. Ajax applications need a built-in process that can improve the quality and performance of Ajax applications. Turc (2019) used Ajax technology supporting dynamic data loading to construct Web applications for Internet of things devices. Ajax has a very promising future since it can improve system performance and optimize user interface. Ajax-existing direct framework AjaxPro can introduce an AjaxPro.2.dll file and directly call the background page method in JavaScript in the front page. However, this framework conflicts with form validation. In addition, Microsoft has also introduced Ajax components. It is essential to add the AjaxControlToolkit.dll file to display relevant controls in the control list. In short, the difference between them is that Jquery is not a technology but a library encapsulated by JS. Ajax is a web development technology to create interactive web applications. The direct web remoting (DWR) framework and the JQuery (JQ) framework can implement Ajax.

### Meta Heuristic Algorithm Optimization

Meta heuristic algorithm can deal with complex problems and find the optimal solution from multiple implementation paths. Genetic algorithm is adopted to design the system structure. Due to the single-point search of the algorithm, the number of transmitted individuals should be small. Adaptive design is adopted, and the specific equation is as follows:


(1) $$m\,i\,z\,e=\left\{ \begin{array}{c} p\,o\,p\,s\,i\,z\,e\,T\,S\\ p\,o\,p\,s\,i\,z\,e\,T\,S^{*}\,A^{*}\,\exp\left(1-g\right)/\left(\max+1-g\right) \end{array}\right.$$

*N* represents the interval of the hierarchical function, *A* represents the fixed coefficient, *max* represents the maximum evolutionary generations, *g* represents the transfer number, and *popsizeTS* represents the population number. Particle swarm optimization (PSO) is used to collect and sort the data. First, the particles are coded. Before making a decision, the particles in the particle swarm are represented as the time and priority of each step. Then, the fitness value of the function is obtained, and the position of each particle is expressed as a decision. PSO algorithm is improved to optimize the inertia weight in the algorithm. The state equation is as follows:


(2) $$w=w_{\min}+\left(w_{\max}-w_{\min}\right)\times \exp\left[-m\,{\left(\frac{t}{t_{\max}}\right)}^{2}\right]$$

*m* represents the factor controlling the smoothness of *w* and *t* change curves, and its value is 3. In the whole process of the algorithm, the mutation mechanism is introduced to prevent the particles in the population from stagnating in the iterative process. If stagnation occurs before the algorithm terminates, the mutation processing is performed. The specific calculation is as follows:


(3) $$if\left(x_{i}\left(t\right)\right)=x_{i}\left(t-1\right)=x_{i}\left(t-2\right)=x_{i}\left(t-n\right)andx_{i}\left(t\neq \eta \right)$$

(4) $$t\,h\,e\,n\,x_{i}\,\left(t+m+1\right)=x_{\min}+r\,a\,n\,d\,\left(0,1\right)\times \left(x_{\max}-x_{\min}\right)$$

η represents the minimum value of fitness function in the whole population, m represents the maximum number of iterations allowed to stagnate, (x_*max*_ − x_*min*_) represents the search range. The maximum number of iterations is set to 1,500; the algorithm runs 20 times and takes the average value as the final result.

## System Design and Environment Setup

### Design of Basic Information Management Module

**FIGURE 1:**
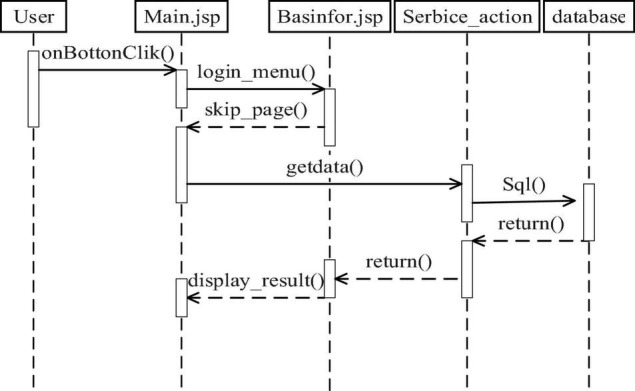
Basic information management module.

The basic information management module is shown in Figure 1. In the basic information management module, the Service_action service class is designed. This class is the most basic information management functions in the class, and the various functions used in the module are stored in the class.

Users log on to the system, enter Main. JSP page, select operation function, select by the login_menu () method, and use the skip_page () method to adjust related pages. Then, users submit a request to get relevant information from the database by getting the data () method and forming an SQL query statement, which queries the corresponding information from the database, and then the system returns the query results (Chen and Chen, 2018). At last, the display_result () method is used to display the query results.

### Design of Teaching Resource Management Module

Teaching resource management is the effective management of video resources in the system. It is mainly responsible for video upload, download, classification, and data statistics.

**FIGURE 2:**
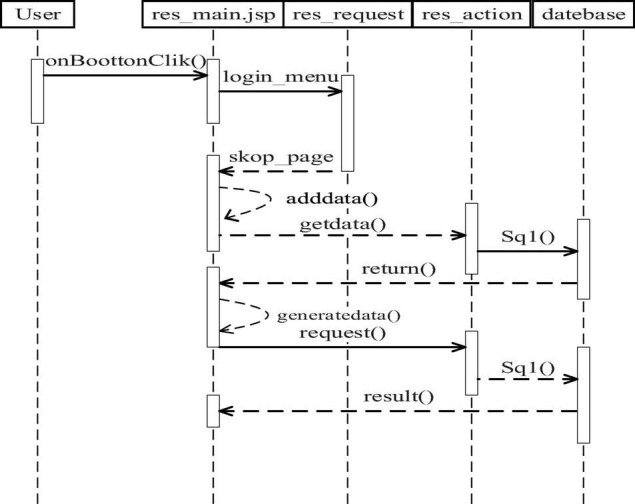
Video upload function timing chart.

The video upload function timing chart is shown in Figure 2. Users log on to the system by user name and password, enter the res_main.jsp page of the system, select the corresponding functional modules and apply the skip_page() method to jump the res_main.jsp to the upload function page. The upload file is added by the adddata () method, the upload request is sent to the system server through res_action, and the corresponding SQL statement is formed (Huang et al., 2016). After inquiring about the relevant information, the information can be processed and returned to the customer page, and the upload file can be submitted. After the upload is completed, the upload result can be returned.

### Design of Communication and Interaction Module

**FIGURE 3:**
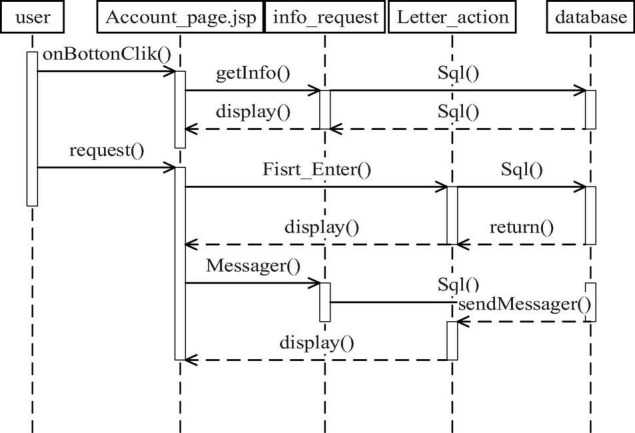
Exchange interaction timing diagram.

Figure 3 is exchange interaction timing diagram. User is the system user, and when the system user logs on to the system, the system will automatically determine whether there is a message or other messages, send the message request through the getInfo () method, form the corresponding SQL statement, query the related statements in the database, return the message list through a list (), and use the display () method to display the list to the user. When the user enters the online communication function, the system obtains speech information of other people from the database by the First_Enter () method, while the sendMessage () method is used to complete the sending of the input message of a user.

### Design of Online Test Module

The online test management module is used to detect a learning situation of students. Teachers judge a learning situation of students by examinations.

**FIGURE 4:**
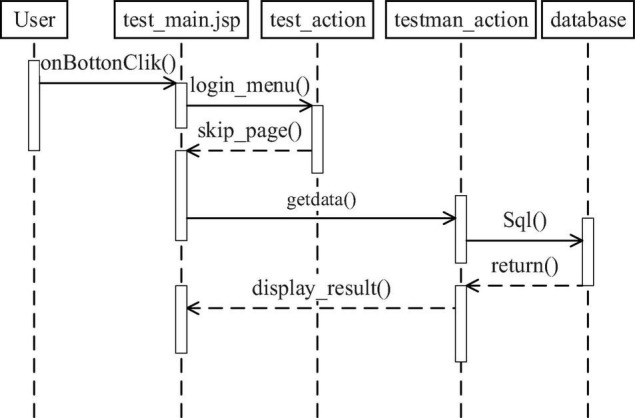
Result query function timing chart.

Figure 4 is result query function timing chart. User is the user of the system. After the user logs in to the system, he or she chooses the functional modules that he or she wants to operate. Then, the login_menu () method is used to submit user requests, and the skip_page () method is used to jump pages. After the user enters the query keyword, the information is sent to the system through the Inputdata () method, the query request is submitted through the GetData () method, and the corresponding SQL query statement is formed to obtain the data information from the system database (Nie, 2018; Pinho et al., 2018). Finally, the query results are displayed on the page by using display_result ().

### Database Design

In the process of designing the database, the multimedia network teaching management system of film and television only explains some entities and introduces the relationship among them because of the actual number of systems.

#### Student Information Table

Table 1 is student information table. Students are one of the main users of this system, and there are many of them. Among them, u_id is the primary key, and the number of students, names, colleges, majors, classes, ID cards, etc., cannot be empty, which must be filled in.

**TABLE 1:** Student information table.

**Field**	**Type**	**Description**	**Can it be empty?**	**Primary key**
u_id	number (8)	Number	No	Yes
u_num	Varchar (10)	Student ID	No	
u_name	Varchar (8)	Name	No	
u_password	Varchar (8)	Password	No	
u_college	Varchar (10)	College	No	
u_pro	Varchar (20)	Major	No	
u_class	Varchar (10)	Class	No	
u_idcard	number (20)	ID number	No	
u_birthday	Date	Date of birth	Yes	
u_phone	number (15)	Telephone	Yes	
u_address	Varchar (10)	Address	Yes	
u_remark	Varchar2(100)	Note	Yes	

#### Test Paper Information Table

Table 2 is test paper information table. Test paper is used to assess students. The attributes of the entity of the paper include the number of the paper, the paper name, the subject, the topic maker, the time of the examination, the upload time, and the time of answering questions. Among them, p_id is the primary key of the enterprise information table, p_num is the foreign key, and the names of other attribute papers, subjects, question makers, examination time, answer time, upload time, and so on cannot be empty.

**TABLE 2:** Test paper information table.

**Field**	**Type**	**Description**	**Can it be empty?**	**Primary key**
p_id	number (8)	Number	No	Yes
p_num	number (8)	Test paper number	No	
p_name	Varchar (10)	Paper name	No	
p_lesson	Varchar (18)	Subject	No	
p_person	Varchar (30)	Topic maker	No	
p_answertime	date	Answer time	No	
p_uptime	date	Upload time	No	
p_remark	Varchar (100)	Note	Yes	

#### Achievement Information Table

Table 3 is achievement information table. The attributes of the achievement information entity include student ID, student name, course, examination question number, entity name, achievement, marker, entry time, notes, etc. Among them, l_id is the primary key of the test paper information table, and the number of students, names, subjects, test paper numbers, test paper names, and scores cannot be empty.

**TABLE 3:** Achievement information table.

**Field**	**Type**	**Description**	**Can it be empty?**	**Primary key**
l_id	Number (8)	Number	No	
u_num	Varchar (8)	Student ID	No	
u_name	Varchar (10)	Name	No	
p_lesson	Varchar (8)	Subject	No	
p_num	number (8)	Test paper number	No	
p_name	Varchar (10)	Paper name	No	
l_results	Int	Score	No	
l_person	Varchar (8)	Judge	Yes	
l_time	Date	Entry time	Yes	
l_remark	Varchar2(200)	Note	Yes	

#### Message Record Table

Table 4 is message record table. Message record table is used to record the information of teachers and students in the process of communication and interaction to facilitate the future inquiry of these messages.

**TABLE 4:** Message record table.

**Field Type**		**Description**	**Can it be empty?**	**Primary key**
r_id	number (8)	Number	No	Yes
r_sender	Varchar (8)	Sender	No	
r_recipient	Varchar (8)	Receiver	No	
r_sendtime	Date	Sending time	No	
r_receiving	Date	Receiving time	No	
r_content	Varchar2(50)	Content	No	
r_note	Varchar (50)	Note		

### System Simulation Test

Software testing is divided from different angles, which can produce different test contents. From the perspective of system analysis, software testing is divided into module testing, function testing, subsystem testing, and overall testing. As for the division from this perspective, the test level increases step by step according to the order from part to whole. Black box testing is used here. The system response time simulation experiment is carried out to verify that the performance index of the system can meet the design requirements. Hardware configuration: central processing unit (CPU): Intel (R) core (TM) 2DuoCPUE8400@3.00 GHz; Memory: 2 GB DDR3 1066 MHz; Hard disk: 320 GB SATA; Graphics card: GeForce GTX 460; Quantity: three sets. Software environment: operating system: LINUX and Windows10; Browsers: Microsoft IE 8, Mozilla FireFox and Google Chrome. Load Runner 7.8 was used as the test tool in the experiment.

## System Implementation and Modules Discussion

### Overall Implementation of the System

System implementation is a process of realizing the system through program code according to the information acquired in the stage of requirement analysis and system design.

**FIGURE 5:**
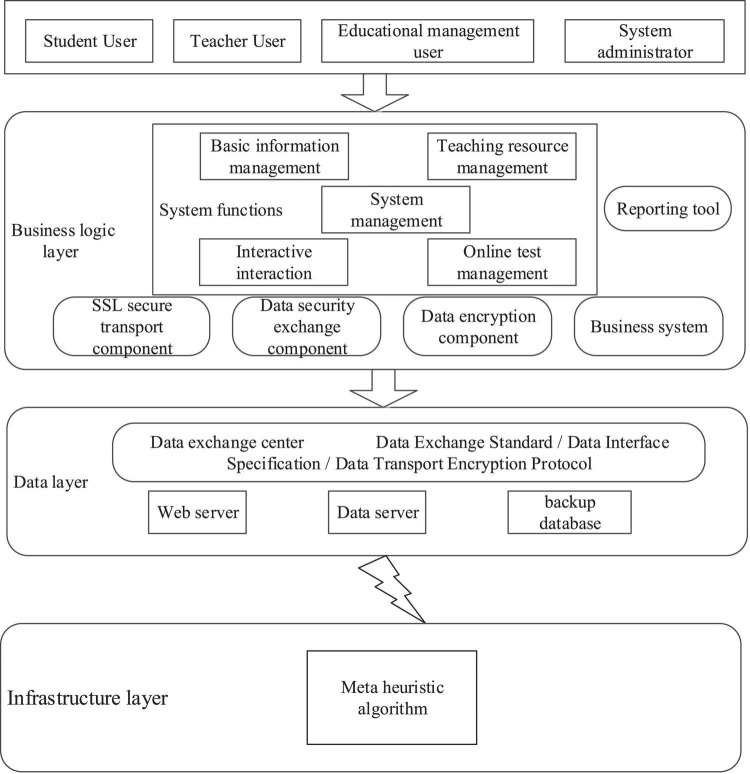
System architecture.

Figure 5 is the architecture of film and television multimedia network teaching management system. The display layer is mainly the interaction layer between users and the system. The business logic layer is the most important part of the whole system. Most of the business logic processing in the system is processed in this layer and embedded in a variety of components, such as SSL (Security Socket Layer) security transmission component, data security exchange component, and data encryption component. Moreover, the business logic layer connects with the business layer on the one hand and the data layer on the other. There are many data interaction protocols and standards in the data layer. In this system, Web server, data server, and backup database are separated. When one database fails, it will not affect other servers to minimize the loss of users.

### Realization of Login Function and Basic Information Module

The user account and the password of the system will be imported from the school management system. The username and the password of the system are the account and the password of the campus management system.

The basic information management module mainly manages some of the most basic information in the system, such as student information, teacher information, curriculum information, etc. The information constitutes the user information of the system. Through the management and maintenance of this information, the system can guarantee the normal service for users.

Users log on to the system, enter the basic information management module to select the student information management function, and the student information management interface will appear in the system page. the related information list of some students will be displayed on the page so that students can be inquired by inputting the names of the students, the name of the institute, and the major they have studied. If the user is not clear about the college where the student is located or major, then by inputting the names of the students, they can find out all the students who have the keyword in the school. Selecting the appropriate operation, they can complete the corresponding modification information modification.

Users log on to the system, enter the system homepage, select the student information management function, and carry out the corresponding operation. Choosing to add the student information function, the new page of student information will appear, and then input the student information that needs to be input, and save the information to the database. Selecting the modification function and clicking on the revised students, the system shows the information of the revised students, then makes the modification of the information, and finally saves the revised information.

**FIGURE 6:**
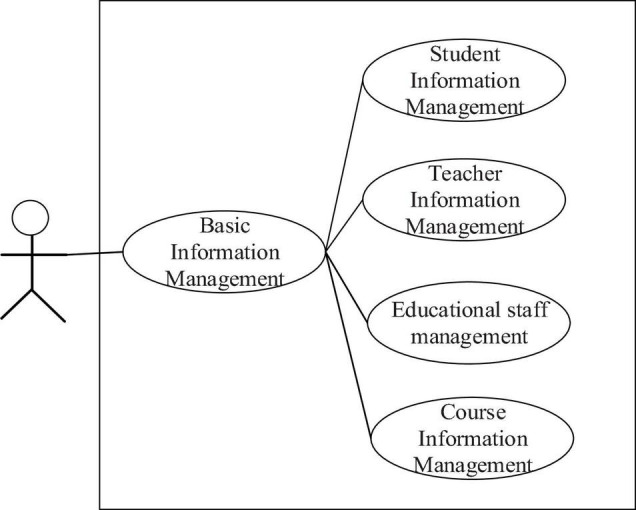
Basic information management of film and television multimedia information management system.

In the course information management, the main aim is to use the teaching course of this system, which needs to be clearly marked. Only the teachers in this course can manage the course information. The basic information management functions of the multimedia information management system for film and television are shown in Figure 6.

### Implementation of Teaching Resource Management Module

Teaching resources are the main function of the system and the core of the system. Teachers can make visible and relevant teaching videos uploaded to the system through uploading function, while students can choose to query relevant teaching resources information according to their needs and download them. At the same time, the data statistics function is set up in this module, which can count the visits and downloads of various teaching resources.

Users log on to the system, enter the system homepage, select the video resource management function, and enter the video function management interface, which shows the video resources that can be managed under the authority of the user. Users can choose the video resources they need to manage. Users log on to the system, select the upload function of video resources, enter the upload page of video resources, input the number, name and course of uploaded video resources, click the browse button, pop up the local file selection interface, select the uploaded video files, click the upload button, and then complete the upload of video resources.

Teachers log on to the system, enter the system homepage, input the relevant information of teaching video, and select the corresponding local video file by browsing function. The system verifies the information input by users and the format and size of the video, and then uploads it to the system to save after verification. When other users log on to the system, they can view the video and download it.

### Implementation of Communication and Interaction Module

The mode of communication and interaction provides an information exchange platform, which is mainly divided into two modes, synchronous communication, and asynchronous communication. At the same time, a discussion area is designed, which provides a great convenience for the learning and communication between teachers and students, and students can learn better.

Users log on to the system, enter the system homepage, select the online communication function, and enter the online communication page, which consists of the information input box, an information display module, and an online person module.

Users log on to the system, enter the system homepage, select the question discussion function, enter the question discussion function page, click the issue button, enter the question publishing interface, and input the relevant information of the discussion problem to complete the issue of discussion. The selected object, after logging on to the question discussion function, will receive the corresponding question discussion information and can release the advice of the issue.

### Implementation of Online Test Management Module

Online test management realizes the detection of the learning situation of students. Teachers upload the test questions to the system. Then, teachers send test notifications to the students who take part in the test. Students log on to the system at a specified time to participate in the test. After completing the test, they submit the answer information.

Teachers log on to the system, enter the system homepage, and select the test notification function. Then, the page jumps to the test notification page, enters the subject and content of the notification, and selects the notification object. The notification object is classes, and, after clicking sending, all members of the class can receive the test notification information.

Student users log on to the system, enter the system homepage, select the performance query function, and enter the student number and test subjects. Then, the page will show all the tests carried out by the subject and select a test score record, and students can query the details of the results. Student users log on to the system, enter the homepage of the system, choose the online test function, choose the test questions, and then begin to answer them. The system displays the contents of the test papers and obtains the test time.

After using the minority films and television multimedia network teaching information management system, the implementation of teaching and learning in the process of multimedia network teaching is comprehensively managed and controlled, which provides a good platform for improving the management level and working efficiency of multimedia network teaching so as to achieve the goal of automation, information, and paperless.

### System Performance Evaluation

**FIGURE 7:**
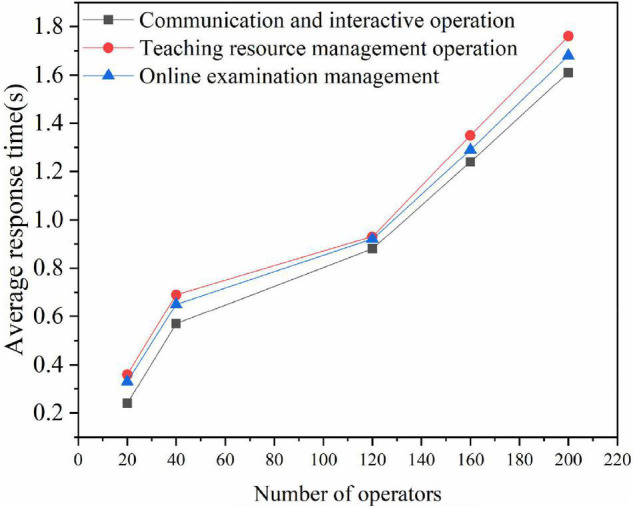
System response delay test results.

The response time given by the system for the operation of the user is also a factor that directly indicates the performance of the system, so it is essential to determine the average response time of all users when using the system. The length of delay time directly affects the sense of experience of the user. After clicking the operation button in the system, if the delay time is short, the user will not even feel waiting for a response, that is, the response result will be obtained immediately after submitting the request. Figure 7 displays a partial result of the test:

For the performance test of the system, different numbers of system concurrency are simulated with the help of 100 computers. In the process of different people accessing and operating the system, the average response time of the system meets the design expectation. Meanwhile, the response time of the system is also within the expected range when the system reaches the maximum number of concurrent users. It indicates that the performance test of the system is good, the system can be put into the network teaching information management, and the good experience of users when using the system can be ensured.

**FIGURE 8:**
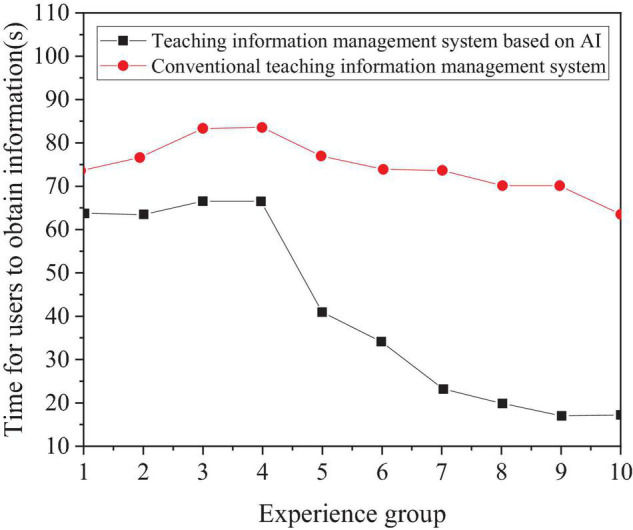
Time curve of users to obtain network teaching information.

Figure 8 is a time curve drawn according to the time when the user obtains teaching resource information, which is obtained from the simulation experiment.

**FIGURE 9:**
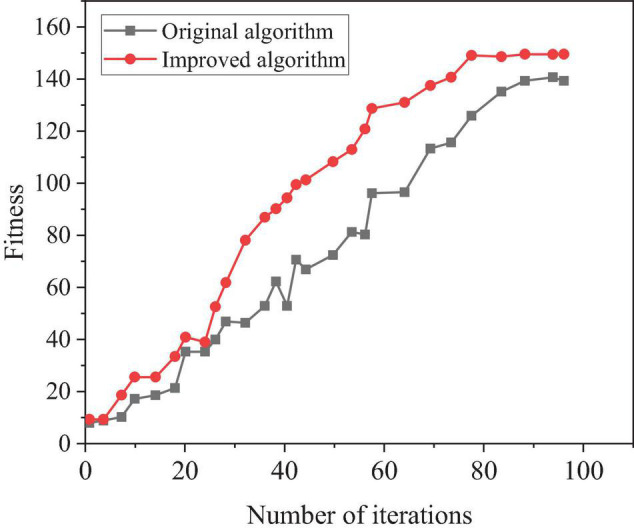
Performance comparison of different algorithms.

The experimental results in Figure 8 show that the time for the conventional management system to obtain teaching resource information is slowly shortening, while the time for the comprehensive management system of English teaching resource information based on AI to obtain teaching resource information decreases rapidly, and the shortest time for obtaining information is 17.5 s. The average of 10 groups of experimental data is calculated. It can be concluded that, compared with the conventional teaching information management system, the time for users of the management system based on AI to obtain teaching resource information is shortened by 36.3 s, which is suitable for the sharing of teaching resource information. Figure 9 displays the performance comparison after improvement.

Figure 9 shows that the performance of the optimized meta heuristic algorithm is significantly better than the original algorithm. In addition, the system can find the optimal solution in a short time under the same number of iterations.

### Student Information Management Function Test Case

The actual test results of student information management are shown in Table 5.

**TABLE 5:** Student information management test results.

**NO**	**Testing procedure**	**Input data**	**Prediction results**	**Confirmation results (Yes/No)**
1	User login film and television multimedia network teaching management system.	User name: 623100235 Password:123456	Enter the interface of film and television multimedia network teaching management system	Yes
2	Trigger “student information management” event	N/A	Enter the student information management interface	Yes
3	Trigger “add” event	N/A	Enter the new student information addition page	Yes
4	Input data	Student No.: 600233 Name: Zhang San Gender: Male Major: Computer Class: TX001 Registered residence: Xinjiang ID number: 650102199510101421 Telephone number: 15160961243 ……	N/A	Yes
5	Managers log in to the system and trigger the “delete” event	N/A	Enter the basic information management interface and display the student information list	Yes

When the teacher manages the student information, the user logs in to the film and television multimedia network teaching management system to trigger the basic information management event, and the page jumps to the basic information management page; then, the user triggers the new event to enter the student information addition interface, inputs the student information, and triggers the save event; the system saves the student information to the database, and the system page returns to the basic information management interface.

## Conclusion

Whether in schools in towns or remote areas, modern teaching methods have greatly alleviated the pressure of traditional teaching methods. The information management system designed for the minority college can make better use of the teaching resources of the college. Compared with other systems, the minority film and television multimedia network teaching information system is characterized by multi-language support. Multimedia network teaching system completes the transmission of educational resources and information through the network. It is based on the network system, combined with multimedia technology, which adds multimedia functions to the system, disseminates various media information through the system, processes media information, and shares information, thus forming an ideal multimedia network teaching environment. Multimedia network teaching system breaks through the limitation of time and space so that students in this school can learn not only in the classroom but also in any place where computers and networks exist. The system also has the function of communication, which can facilitate the communication between students and between students and teachers and provide an effective teaching management tool for teachers. To sum up, the development of the multimedia system plays a positive role in the development of regional education for ethnic minorities.

Based on the in-depth analysis of the similar multimedia network teaching management system of film and television, the development process and key points of the system are clarified. Then, go deep into colleges and universities; communicate with teachers, students, and teaching staff; and determine their needs in multimedia network teaching. Design and complete the system framework, and use the related system design technology to improve the overall architecture and functional architecture of the system. In order to make the function of the system as perfect as possible, the system is tested comprehensively and the omissions are corrected. The multimedia network teaching management system for film and television has the function of communication, which can realize the communication between students and between students and teachers, and also provides a set of teaching management tools for teachers.

In general, while designing a multimedia network teaching management system, Python is more suitable for the development of this system. It has excellent versatility and high efficiency. Python can provide necessary and effective processing tools in the whole process. Each step has a special tool library. Python contains multiple powerful statistical and mathematical tools, such as Pandas, Numpy, Matplotlib, SciPy, and scikit-learn. Besides, it also includes advanced deep learning tools, such as Tensorflow and PyBrain. Furthermore, Python is recognized as the basic language of AI and machine learning, and there is a close intersection between data science and AI. A system developed by Python can be transplanted to other platforms. In this study, by designing a multimedia network teaching management system, the needs of college students and teachers in network teaching are met. Especially, the classification of minority languages makes the learning of students no longer limited by time, space, and language, which is more convenient to obtain teaching resources to meet the learning needs. Multimedia network teaching management system has a wide range of points. The research significance is to change the data management means in the traditional teaching work, enhance the interaction of multimedia information, make the multimedia teaching resources really play its teaching value in the teaching process, and provide an open and shared learning interactive platform for teachers and students. At present, there are still many areas that need further improvement in the system. For example, the logic design of the application logic layer in the three-tier structure is not perfect enough and should be further refined. Data acquisition has not yet reached the level of full automation. In the future, it is necessary to continue to study the programming interface of the equipment to achieve full automation acquisition.

## Data Availability Statement

The original contributions presented in the study are included in the article/supplementary material, further inquiries can be directed to the corresponding author.

## Ethics Statement

The studies involving human participants were reviewed and approved by Southwest Minzu University Ethics Committee. The patients/participants provided their written informed consent to participate in this study. Written informed consent was obtained from the individual(s) for the publication of any potentially identifiable images or data included in this article.

## Author Contributions

The author confirms being the sole contributor of this work and has approved it for publication.

## Conflict of Interest

The author declares that the research was conducted in the absence of any commercial or financial relationships that could be construed as a potential conflict of interest.

## Publisher’s Note

All claims expressed in this article are solely those of the authors and do not necessarily represent those of their affiliated organizations, or those of the publisher, the editors and the reviewers. Any product that may be evaluated in this article, or claim that may be made by its manufacturer, is not guaranteed or endorsed by the publisher.
